# 

**Published:** 1907-12

**Authors:** 


					J?11 *S?J Contents.
Page
Syphilis.
By Henry Waldo, M.D.
289
Some Remarks onJ^uaUknajsthesia as based upon the Personal
.-Observation - of Thirty Cases.
By Ernest W. Hey Groves,
M.S. Lond., F.R C.S.
305
The Value of Compression of the Aorta in the Treatment ot Post-
Partum Hemorrhage.
I1. Percy Elliott, M.is.
3io
A Case of Generalised Sarcoma, with Blood Changes.
By F. G.
Bushnell, M.D.
321
Proportional Representation and the Comparison ot Kadiographs.
By William Cotton, M.A., JV1 ^U., u.t'.ti.
326
Public Health in Bristol, 1906-7.
By D. S. Davies, MD.
336
Progress of the Medical Sciences
Medicine.
J. Michell Clarke, M.A., M.D.
34i
Surgery.
James Swain, M.S., M.D.
344
Psychiatry.
F. St. John Bullen
34?
Reviews of Books
353
Editorial Notes
3&?
Notes on Preparations for the Sick
374
?He Library of the Bristol Medico-Chirurgical Society ... ... 379
Meetings of Societies
382
Local Medical Notes
3?3

				

